# The Role of Nutritional and Inflammatory Indices in Predicting Prognosis in Older Adults Undergoing Radiotherapy for Lung Cancer: NIRT-LC Study

**DOI:** 10.3390/jcm15072756

**Published:** 2026-04-06

**Authors:** Ilyas Akkar, Harun Demir, Ibrahim Babalioglu, Muhammet Cemal Kizilarslanoglu

**Affiliations:** 1 Division of Geriatrics, Department of Internal Medicine, Konya City Hospital, University of Health Sciences Türkiye, Konya 42020, Türkiye; drcemalk@yahoo.com.tr; 2 Department of Radiation Oncology, Konya City Hospital, University of Health Sciences Türkiye, Konya 42020, Türkiye; alidmr198@gmail.com (H.D.); drbabalioglu@hotmail.com (I.B.)

**Keywords:** older adults, lung cancer, survival, radiotherapy, immunonutritional indices

## Abstract

**Background/Objectives:** The aim of this study was to identify which pre-radiotherapy (RT) immunonutritional indices best predict mortality and overall survival in geriatric patients with lung cancer (LC). **Methods:** This retrospective single-center study included LC patients aged ≥ 65 years who underwent RT between August 2020 and December 2024. Clinical records and laboratory data obtained within 14 days before RT were used to calculate immunonutritional indices. Survival and subgroup analyses evaluated prognostic significance. **Results:** Among the 174 patients included in the study, the median age was 69 years, and the median follow-up after RT was 8 months. Inflammatory indices were higher among non-survivors, whereas nutritional indices were lower (all *p* < 0.05). The ROC curve analyses identified the Prognostic Nutritional Index (PNI), Geriatric Nutritional Risk Index (GNRI), and CALLY (CRP–Albumin–Lymphocyte Index) as the strongest predictors of mortality (AUCs > 0.700). In adjusted Cox models, CALLY (HR = 0.652), PNI (HR = 0.939), and GNRI (HR = 0.950) were independently associated with reduced mortality risk. **Conclusions**: In older adults with LC undergoing RT, pre-treatment immunonutritional indices were independently associated with overall survival. Lower inflammatory burden and higher nutritional scores were linked to improved outcomes. These indices were associated with mortality before RT across LC types, independent of disease stage. Among them, CALLY, PNI, and GNRI showed the strongest associations with mortality, suggesting that these markers may be promising candidates for pre-RT risk assessment. However, further validation in prospective cohorts is required before routine clinical implementation.

## 1. Introduction

LC is the most commonly diagnosed malignancy [1]. In 2022 alone, approximately 2.5 million new cases were reported, accounting for over 800,000 deaths [2]. The treatment of LC is often complex and involves multiple therapeutic modalities such as surgery, RT, and systemic therapies [3]. RT is an important treatment modality that may be applied across a range of disease stages and patient performance statuses, depending on individual clinical characteristics [4]. It remains a cornerstone of cancer treatment, with over half of the patients receiving RT [5]. Among geriatric patients, RT is often preferred because of its efficacy and low systemic toxicity [6].

Malnutrition is common among older patients with cancer, primarily due to reduced nutritional intake and cancer-related side effects [7]. Nutritional status influences treatment choices and is associated with complications and mortality [8]. Maintaining adequate and balanced nutrition is important for cancer patients, as cancer-related cachexia or sarcopenia, characterized by muscle loss and increased protein catabolism, can impair the systemic immune response [9,10].

RT modulates the immune system by altering the tumor microenvironment, inducing cytokine/chemokine release, promoting leukocyte infiltration, and enhancing tumor cell susceptibility to immunogenic death [11]. Immune-nutritional status and RT-induced inflammation may critically influence the disease course and outcomes; however, survival and toxicity show substantial interpatient variability [12]. However, further research is needed to more clearly characterize the factors associated with treatment-related toxicities.

Numerous studies have investigated predictive markers in LC. However, older adults represent a heterogeneous population characterized by age-related changes such as immunosenescence, chronic low-grade inflammation (“inflammaging”) [13], and a higher prevalence of malnutrition, sarcopenia, and frailty [14]. These factors may influence both the host inflammatory response and nutritional status, which are the key components of immunonutritional indices. To our knowledge, studies that simultaneously evaluate both inflammatory and nutritional indices within the same cohort of older adults to assess their prognostic value in LC remain limited.

Therefore, this study aimed to determine which pre-treatment immunonutritional indices better predict prognosis in geriatric patients receiving radiotherapy for LC.

## 2. Materials and Methods

### 2.1. Patient Selection

**Figure 1:**
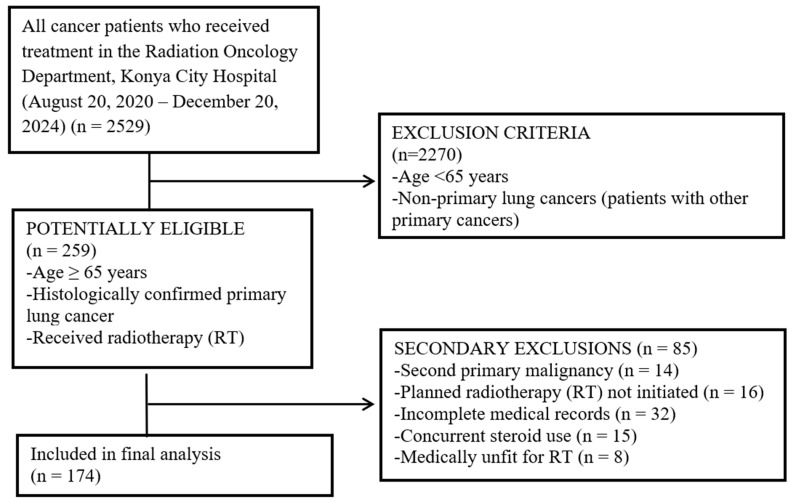
Flow chart identifying the patients.

In this retrospective study, patients aged ≥ 65 years with LC who received RT at the Department of Radiation Oncology, Konya City Hospital, between 20 August 2020 and 20 December 2024, were included. Among the 2529 patients screened, the majority were excluded due to age < 65 years or the presence of non-primary LC, resulting in a final cohort of 174 eligible patients. The enrollment flowchart detailing the exclusion criteria is presented in Figure 1.

Inclusion Criteria:

1. Pathologically confirmed LC diagnosis.
2. Age ≥ 65 years.
3. The patients who were expected to survive at least 3 months were included. This criterion was based on clinical judgment documented in physician notes during treatment planning, including multidisciplinary tumor board evaluations. Patients with a documented expected survival of less than 3 months due to advanced disease, poor performance status, or other clinical factors were not considered eligible for radiotherapy.
4. Documented blood test results were obtained within 14 days preceding the start of RT.
5. Receiving RT for LC at any stage.
6. Full availability of clinical and laboratory parameters.

Exclusion Criteria:

1. Age < 65 years.
2. Not receiving RT.
3. Use of systemic corticosteroids.
4. Presence of hematologic or autoimmune disorders.
5. Diagnosis of a second primary malignancy.
6. Patients with active infection receiving treatment at RT initiation were excluded.

### 2.2. Patient Characteristics

Clinical data were extracted from patient medical records and the hospital information system, including electronic records, physician notes, and consultation reports. The collected variables included age, sex, body mass index (BMI), chronic comorbidities, cancer type and site of involvement, history and type of surgery, prior systemic chemotherapy, RT characteristics (type, total dose, and number of fractions), and RT adherence. Treatment modality and intent (curative or palliative) were also recorded. ECOG performance status was obtained from performance status notes in the patient records [15].

### 2.3. Laboratory Analysis and Calculation of Immunonutritional Indices

Within 14 days prior to the initiation of RT, the most recent laboratory results were evaluated, including hemoglobin, white blood cell (WBC), platelet, neutrophil, lymphocyte, and monocyte counts, albumin, creatinine, and C-reactive protein (CRP). Various inflammatory and immunonutritional indices were calculated based on these parameters. The relevant ratios were derived from values obtained using automated hematology analyzers (Sysmex Corporation, Kobe, Japan).

The indices and their calculation methods are summarized below:

- Neutrophil Lymphocyte Ratio (NLR): neutrophil count/lymphocyte count.
- Platelet Lymphocyte Ratio (PLR): platelet count/lymphocyte count.
- Monocyte Lymphocyte Ratio (MLR): monocyte count/lymphocyte count.

The Advanced Lung Cancer Inflammation Index (ALI) is an inflammation-related prognostic marker in lung cancer. It is calculated as the product of BMI and serum albumin (g/dL) divided by the NLR [16]. Systemic Immune-Inflammation Index (SII): represents systemic inflammation and immune activity in patients with cancer and has been correlated with prognosis. SII = (platelets × neutrophils)/lymphocytes [17].

Hemoglobin, Albumin, Lymphocyte, Platelet (HALP) score: This is a useful prognostic biomarker in cancer patients [18]. It was calculated as follows: HALP = [hemoglobin × albumin × lymphocyte count]/platelet count.

CALLY: This is an index that integrates both inflammatory and nutritional status [19]. The following formula was used for the calculation: CALLY = (albumin × lymphocyte)/(CRP × 10^4^) [20].

GNRI: This scale assesses nutrition-related morbidity and mortality risk in older adults [21]. GNRI = 1.489 × serum albumin (g/L) + 41.7 × actual body weight (kg)/optimal body weight (kg), where optimal body weight corresponded to a BMI of 22 kg/m^2^, calculated based on the patient’s height [22].

PNI: This reflects the immunonutritional status of patients with malignancy. The following formula was used for the calculation [23]. PNI = [10 × albumin (g/dL)] + [0.005 × total lymphocyte count].

Given that these immunonutritional indices are derived from shared components reflecting systemic inflammation and nutritional status, they are inherently mathematically overlapping and biologically correlated [24].

### 2.4. Clinical Follow-Up Data Related to the Radiotherapy Process

Toxicities observed during the RT process, hospital admissions, and their potential associations with RT were monitored and recorded using the hospital information system. Additionally, the dates of the last follow-up and the current clinical status of each patient were retrieved from the hospital database. The death dates of the deceased patients were verified using hospital records.

The primary endpoint of the study was overall survival (OS), defined as the time from the initiation of RT to death from any cause or last follow-up.

### 2.5. Toxicity Assessment

T-related acute toxicities were evaluated and graded according to the RTOG Acute Radiation Morbidity Scoring Criteria [25]. Toxicities involving the skin, upper gastrointestinal tract, esophagus, and hematologic parameters were classified as grades 1–4. RT-related toxicity was defined as clinically significant acute toxicity occurring during RT.

### 2.6. Statistical Analysis

After evaluating the normality of continuous variables using the Kolmogorov–Smirnov test, numerical data were reported as mean ± standard deviation or median (min–max). Categorical variables were presented as numbers (n) and frequencies (%). The Student’s *t*-test or Mann–Whitney U test was used to compare numerical variables between groups based on their distribution patterns. Associations between clinical and immunonutritional variables and mortality were evaluated using univariable analyses, and variables with *p* < 0.10 were entered into multivariable models. The Cox regression analysis was done to find out the independently related parameters for mortality.

For continuous predictors independently associated with mortality, optimal cut-off values were determined using ROC curve analysis, and AUCs and *p*-values were calculated. Survival probabilities and median survival times were estimated using the Kaplan–Meier method and compared with the log-rank test. Unless otherwise stated, *p* < 0.05 was considered statistically significant. The IBM SPSS Statistics (version 27.0; IBM Corp., Armonk, NY, USA) program was used for the statistical analyses. This study was a retrospective observational study that included all consecutive eligible patients during the study period. Therefore, no a priori sample size calculation was performed, as the sample size was determined by the available cohort (estimated as 100%). However, a post hoc power analysis was conducted for the study. First, we designed the Cox regression models separately for each index. Therefore, the adjusted Cox regression models contained six variables (index, age, sex, lung cancer type, presence of metastasis, and BMI). In our cohort of 174 patients with 121 events (mortality), the events-per-variable (EPV) ratio was approximately 20, exceeding the commonly recommended minimum threshold of 5–10 events per variable and supporting the stability and reliability of the regression estimates. On the other hand, when we have performed a post hoc power analysis using [G*Power 3.1.9.4 version], based on the observed event rate (121 events/174 patients) and effect size, the study had approximately 86.4% power to detect a hazard ratio of 1.5 or greater at a two-sided α level of 0.05.

## 3. Results

### 3.1. Patient Characteristics and Clinical Profile

Patients had a median follow-up of 12 months (range, 0–69 months) from the time of malignancy diagnosis and 8 months (range, 0–43 months) following RT. Among the study population, 121 patients (69.5%) died during follow-up. The median age was 69 years (range, 65–87). Sex distribution was comparable between survivors and non-survivors; 78.7% (n = 137) had ≥2 comorbidities. The most common conditions were non-small cell lung cancer (NSCLC) (81.6%), hypertension (42.5%), coronary artery disease (24.1%), and diabetes mellitus (23%). At diagnosis, 57.3% (*n* = 100) of the patients had metastatic disease, with bone being the most frequent site of metastasis (42%, *n* = 73).

Regarding RT, the majority of patients received palliative RT (57.5%, *n* = 100), followed by definitive and adjuvant RT (42.5%, *n* = 74). During RT, 32.8% (*n* = 57) of patients required hospitalization. RT compliance was high, with 90.2% (*n* = 157) completing the planned treatment, and RT-related toxicity was reported in 22.4% of patients (*n* = 39). As part of oncological management, 16.7% (*n* = 29) of patients underwent surgery, while 81.6% (*n* = 142) received systemic therapy. Notably, the study cohort was heterogeneous, including patients with different lung cancer subtypes (SCLC and NSCLC), disease stages (localized, locally advanced, and metastatic), and treatment indications. All patients included in the study had received RT, reflecting real-world clinical practice.

### 3.2. Comparison by Mortality Status

The baseline characteristics were compared between survivors and non-survivors at the end of the follow-up period. Non-survivors were significantly older and had a lower BMI, poorer ECOG performance status, and more advanced disease stages compared to survivors; moreover, they were less frequently administered systemic anticancer therapy or subjected to surgical intervention. In contrast, the rates of palliative RT and hospitalization during RT were significantly higher among non-survivors (*p* = 0.002, *p* = 0.007, *p* = 0.001, *p* < 0.001, *p* = 0.004, *p* = 0.022, *p* < 0.001, *p* = 0.001, respectively). Comorbidities were similar between groups (*p* > 0.05).

### 3.3. Laboratory Parameters

Non-survivors had lower lymphocyte and albumin levels and higher CRP levels than survivors (*p* < 0.001, *p* < 0.001, and *p* = 0.010, respectively). Other laboratory parameters were similar (*p* > 0.05). The detailed laboratory findings are presented in Table 1.

Immunonutritional indices (NLR, PLR, MLR, SII, ALI, HALP, CALLY, PNI, and GNRI) differed significantly between survivors and non-survivors. Non-survivors had higher inflammatory indices (NLR, MLR, PLR, and SII) and lower nutritional indices (ALI, HALP, CALLY, PNI, and GNRI) (Table 2).

### 3.4. Cox Regression Models and ROC Curve Analyses

Cox proportional hazards regression was used for time-to-event analyses to assess associations between immunonutritional indices and post-RT mortality. To avoid multicollinearity among the interrelated immunonutritional indices, separate Cox proportional hazards models were constructed for each index rather than a single combined model. For each index, an initial univariable (crude) analysis was performed, followed by models adjusted for clinically relevant covariates, including age, body mass index (BMI), sex, type of lung cancer (SCLC vs. NSCLC), and presence of metastasis.

In the adjusted model, NLR, PLR, MLR, SII, HALP, CALLY, PNI, and GNRI remained significant predictors of mortality (all *p* < 0.05). On a continuous scale, higher NLR (HR = 1.045), PLR (HR = 1.002), MLR (HR = 3.199), and SII (HR = 1.017) were associated with an increased risk of death, whereas higher HALP (HR = 0.844), CALLY (HR = 0.652), PNI (HR = 0.939), and GNRI (HR = 0.950) were associated with a reduced risk (Table 3).

We also applied a Benjamini–Hochberg correction as a sensitivity analysis, which did not significantly change the significance of the main results (*p*-values) shown in the adjusted regression table. Therefore, in our dataset, the first eight *p*-values are considered statistically significant at a 5% “False Discovery Rate” (FDR). The last *p*-value (0.151) (of the ALI score) remains not significant after this correction.

**Figure 2:**
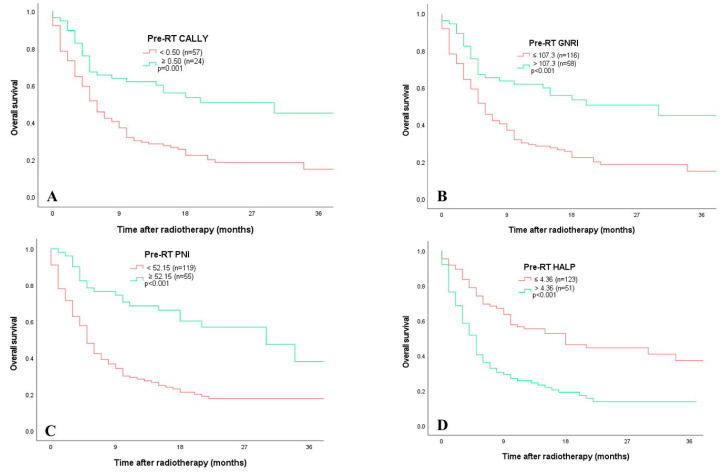
Kaplan–Meier curves for overall survival (OS) according to baseline immunonutritional indices in patients with lung cancer receiving radiotherapy (*n* = 174). Log-rank test. ((**A**) 4 months vs. 22 months), ((**B**) 6 months vs. 30 months), ((**C**) 5 months vs. 34 months), ((**D**) 5 months vs. 30 months).

**Figure 3:**
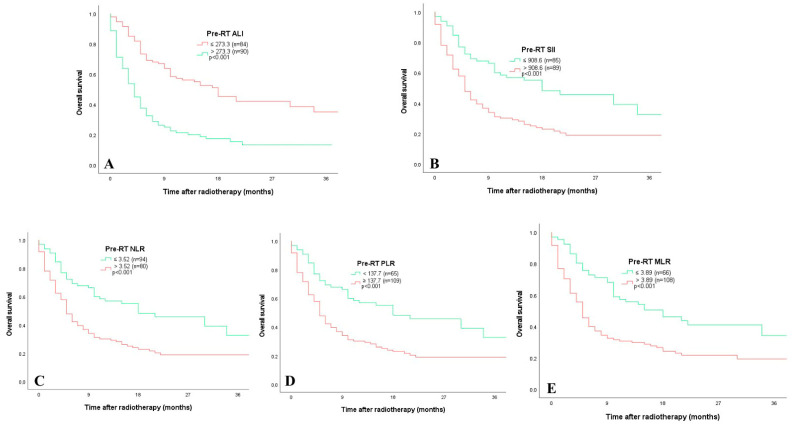
Kaplan–Meier curves for overall survival according to baseline immunonutritional indices in patients with lung cancer receiving radiotherapy (*n* = 174). Log-rank test. ((**A**) 4 months vs. 18 months), ((**B**) 5 months vs. 18 months), ((**C**) 4 months vs. 18 months), ((**D**) 5 months vs. 18 months), ((**E**) 5 months vs. 18 months), (MLR level × 10 was presented in the figure).

ROC analysis was used to assess the prognostic value of the indices for post-RT mortality. Among inflammatory markers, the optimal cut-off values derived from ROC curves were as follows: NLR > 3.52 (AUC = 0.669, *p* < 0.001), PLR ≥ 130.76 (AUC = 0.650, *p* = 0.001), MLR > 0.389 (AUC = 0.622, *p* = 0.007), and SII > 908.6 (AUC = 0.637, *p* = 0.002). Regarding nutritional indices, the optimal thresholds for mortality were determined as follows: CALLY < 0.50 (AUC = 0.728, *p* = 0.002), ALI ≤ 273.3 (AUC = 0.695, *p* < 0.001), HALP score ≤ 4.36 (AUC = 0.685, *p* < 0.001), GNRI ≤ 107.3 (AUC = 0.714, *p* < 0.001), and PNI < 52.15 (AUC = 0.736, *p* < 0.001). Based on the optimal cut-off values determined for each index, overall survival (OS) was compared among subgroups using Kaplan–Meier curves. Statistically significant differences in the EFS were observed across all indices (*p* < 0.001 for all). Patients with lower pre-RT values for the CALLY index (<0.50), ALI (≤273.3), HALP score (≤4.36), GNRI (≤107.3), and PNI (<52.15) exhibited significantly shorter EFS than those with higher values. Similarly, elevated inflammatory indices such as SII (>908.6), NLR (>3.52), PLR (≥130.76), and MLR (>0.389) were significantly associated with poorer survival outcomes (Figure 2 and Figure 3).

## 4. Discussion

In the present study, we evaluated the prognostic significance of several pre-RT inflammatory and immunonutritional biomarkers, including NLR, PLR, MLR, SII, ALI, HALP, CALLY, PNI, and GNRI, in older patients with LC undergoing RT. Our results suggest that these indices are associated with survival independent of tumor stage and histological subtype. Among them, GNRI, PNI, and CALLY appeared to demonstrate relatively stronger prognostic associations. From a clinical perspective, the assessment of nutritional and inflammatory status prior to RT may meaningfully contribute to clinical decision-making in geriatric patients. Easily applicable indices based on routine laboratory parameters, such as the GNRI, PNI, and CALLY, allow for rapid and practical identification of patients with increased frailty and a higher risk of complications before treatment initiation. In patients with low index values, targeted supportive strategies, including closer clinical monitoring, planning enteral nutritional support, and evaluation of potential sources of infection, may be implemented before and during RT. This holistic approach may help to improve RT tolerance and support treatment continuity. Tumor-derived inflammatory cytokines contribute to weight loss and cachexia, whereas malnutrition, infiltration of inflammatory mediators, and immune dysregulation collectively accelerate disease progression [26]. The aforementioned vicious cycle disrupts nutritional inflammatory homeostasis, thereby increasing the risk of mortality. These observations suggest that incorporating GNRI, PNI, and CALLY into pre-RT evaluations may provide valuable prognostic information to guide individualized supportive care in this vulnerable population. However, prospective validation studies are required before these indices can be established as standard adjuncts to clinical decision-making. These indices were calculated from anthropometric measurements (height and body weight) and laboratory parameters obtained within 14 days before RT initiation, including WBC, hemoglobin, platelet, neutrophil, monocyte, and lymphocyte counts, as well as CRP and serum albumin levels.

The study population was predominantly male. Similarly, in our country, the prevalence of lung disease in this age group is also higher among men [27]. Similarly, the majority of older adult patients and those undergoing RT were male, a finding consistently reported at high rates in the literature. For example, one study reported a male proportion of 84.3% [28], while another documented this rate as 70% [29]. Smoking is a well-established risk factor for LC [30]. The higher proportion of males observed in our study may be attributed to the substantially lower prevalence of smoking among women.

In our study, treatment compliance was high, with 90.2% of patients completing the planned RT protocol without interruption, consistent with previous reports demonstrating that older patients generally tolerate RT well [31,32,33]. In stage III NSCLC, the incorporation of 18F-FDG PET-CT into RT planning may further improve treatment accuracy by enabling precise delineation of tumor extent and nodal involvement [34]. By reducing geographic misses and potentially limiting unnecessary radiation exposure to surrounding tissues, this approach may also contribute to better tolerability and, consequently, enhanced treatment compliance.

The majority of patients (81.6%) had NSCLC, which aligns with its known predominance among LC subtypes [35,36]. These findings support the applicability of our results to a representative older lung cancer population.

High NLR, PLR, and MLR levels are correlated with poor prognosis in LC [37,38,39]. Our findings are consistent with the existing literature and further support these associations. The significantly lower GNRI and PNI in non-survivors align with the established links between malnutrition, mortality, and poor prognosis [40]. These findings support routine pre-RT nutritional assessment and subsequent support for patients with LC.

In our study, PNI, NLR, and MLR demonstrated significant prognostic value; non-survivors exhibited markedly lower PNI values along with higher NLR and MLR values. Lymphocyte counts were significantly lower in the non-survivors than in the survivors. PNI, NLR, and MLR have been associated with poor prognosis across various cancer types [41,42], and our findings support and extend these observations to older adult patients with LC. Lymphopenia may reflect a reduced ability of the immune system to mount an effective antitumor response and, through mechanisms such as suppression of T cell–mediated antitumor activity and increased immunosuppression within the tumor microenvironment, could lead to worse clinical outcomes. In our study, we evaluated geriatric patients with small-cell lung cancer (SCLC) and NSCLC. Most patients were at stage IV and had received systemic therapy, while a subset had undergone surgical treatment.

Existing evidence suggests that low PNI is associated with higher mortality [40]. Similarly, our study assessed the prognostic value of the pre-RT PNI in geriatric patients with LC. ROC curve analysis identified a PNI value of 52.15 as the optimal cutoff point. A PNI below this threshold significantly predicts increased mortality. These findings highlight the need to assess nutritional and inflammatory status before initiating RT in geriatric patients with LC. In cases with low PNI (<52.15), pre-treatment optimization of nutrition and inflammation, when feasible, may improve treatment tolerance and outcomes. Integration of multidisciplinary nutritional support interventions within the therapeutic paradigm of this vulnerable demographic may augment radiotherapeutic efficacy whilst attenuating mortality risk. However, this cutoff value should be considered cohort-specific, and further studies are needed to validate its applicability in different populations.

A previous study linked a GNRI value above 98 to improved survival in advanced-stage NSCLC patients [43]. In another study, the cutoff value for the GNRI was determined to be 108.15 [44]. In our study, the GNRI cutoff value that best predicted the prognosis of LC patients receiving RT was 107.3. The variability in the GNRI cutoff values across studies may be attributed to differences in the inclusion of specific lung cancer subtypes. All patients with LC who received RT were included in our study. While previous studies have demonstrated that the GNRI assessed at the time of diagnosis may predict mortality, our findings indicate that the GNRI evaluated before RT serves as a significant prognostic marker in this patient population. Nevertheless, this cutoff should be interpreted as specific to our study cohort and requires external validation.

The CALLY index has been shown to correlate with prognostic outcomes in cancer patients across multiple studies [45,46]. Liu et al. demonstrated that a CALLY score < 1.32 predicted worse prognosis in NSCLC patients [47]. In our study, ROC analysis revealed that patients with LC who had a CALLY score above 0.50 exhibited better prognostic outcomes. In this study, the prognostic utility of the CALLY score was evaluated in patients with LC before RT, and the cut-off value was determined as 0.50. Scores equal to or above this threshold were associated with a favorable prognosis, whereas lower scores indicated poorer outcomes. The relatively low cutoff may be related to the inclusion of all lung cancer subtypes, the older age of the patients, and the fact that the parameters were measured prior to RT rather than at diagnosis, potentially reflecting disease progression or prior treatments such as chemotherapy. Consistent with previous studies, a higher CALLY score remains a potential indicator of favorable prognosis in this setting. However, similar to other indices, this cutoff value is cohort-dependent and should be validated in independent and prospective cohorts before broader clinical application.

The biomarkers investigated in this study serve as practical, accessible, and cost-effective indicators of the systemic inflammatory response and nutritional status. As the clinical roles of these markers have become more clearly defined and given that most radiation oncologists worldwide have easy access to these tests, their potential for use in routine clinical practice is strengthened. These biomarkers can provide valuable information for predicting patient prognosis and overall survival.

### Strengths and Limitations

One of the main strengths of this study is its comprehensive evaluation of multiple inflammatory and immunonutritional indices in a geriatric population with LC. Focusing specifically on older adults provides clinically relevant insights for a population that is frequently underrepresented in oncologic prognostic studies. This approach enabled an integrated analysis of systemic biomarkers associated with prognosis, offering a more holistic understanding of their potential clinical relevance. Another important strength is the inclusion of patients with different histological types and stages of lung cancer, whereas many previous studies have focused on more restricted populations, such as patients with either small-cell or non-small-cell lung cancer or those stratified only by metastatic status. Several limitations should be acknowledged. First, the retrospective single-center design may limit the generalizability of the findings. Second, the study population was heterogeneous in terms of disease stage and treatment intent, which may have influenced the observed associations and should be considered when interpreting the results. However, to minimize this worst effect, we adjusted the regression models for age, body mass index (BMI), sex, type of lung cancer (SCLC and NSCLC), and presence of metastasis. Third, assessing immunonutritional indices at only one pre-RT time point precluded evaluation of their dynamic changes during treatment. Fourth, the lack of baseline measurements at the time of diagnosis limited our ability to assess their full prognostic potential. Additionally, multiple biomarkers were evaluated in parallel, which may increase the risk of type I error despite their biological interrelatedness. Finally, because many of the evaluated indices share overlapping components and are inherently correlated, they were entered into separate regression models to minimize potential multicollinearity.

An additional limitation relates to the inclusion criterion of an expected survival of at least 3 months, which was retrospectively determined based on physician documentation and clinical decision-making. This approach is inherently subjective and may have introduced selection bias by preferentially including patients with better baseline clinical status. Consequently, the study population may underrepresent the frailest patients with the poorest prognosis, thereby limiting the generalizability of the findings. Furthermore, this criterion may be associated with potential immortal time or selection bias, which should be considered when interpreting the results.

Future multicenter prospective studies with serial measurements from diagnosis through treatment completion are needed to better clarify the clinical utility of these biomarkers.

## 5. Conclusions

In older adults with LC undergoing RT, lower pre-RT levels of NLR, PLR, MLR, and SII and higher HALP, CALLY, GNRI, and PNI scores were associated with improved overall survival. These associations were observed regardless of age, BMI, sex, lung cancer type, and metastatic status. Among the evaluated biomarkers, CALLY, GNRI, and PNI demonstrated relatively better discriminative performances. Routine assessment of these markers before RT may help identify high-risk patients, guide survival estimation, and facilitate timely nutritional or supportive interventions. However, these findings should be interpreted with caution, and prospective validation studies are needed before these biomarkers can be recommended for routine clinical use.

## Figures and Tables

**Table 1:** Comparison of Baseline Demographic, Clinical, Disease-Related, and Radiotherapy Characteristics of the Study Population according to mortality status.

Parameters	Total (*n* = 174)	Survivors (*n* = 53)	Non-Survivors (*n* = 121)	*p*-Value
**Baseline characteristics**				
Age, years, median (min-max)	69 (65–87)	67 (65–77)	70 (65–87)	0.002
BMI, kg/m^2^, median (min-max)	23.76 (15.39–37.02)	24.8 (18.44–37.02)	23.36 (15.39–33.33)	0.007
Male sex, n (%)	160 (92)	47 (88.67)	113 (93.4)	0.307
**Comorbidities, *n* (%)**				
Diabetes Mellitus	40 (23)	16 (30.5)	24 (19.8)	0.135
Hypertension	74 (42.5)	26 (49.1)	48 (39.7)	0.246
Coronary artery disease	42 (24.1)	14 (26.4)	28 (23.1)	0.642
Chronic obstructive pulmonary disease	57 (32.8)	20 (37.7)	37 (30.6)	0.355
Cerebrovascular disease	7 (4)	2 (3.8)	5 (4.1)	0.912
Number of comorbid diseases	2 (1–6)	3 (1–6)	2 (1–6)	0.389
**Lung Cancer-Related Characteristics, *n* (%)**				
NSCLC	142 (81.6)	47 (88.67)	95 (78.5)	0.111
ECOG Performance Status = 1, n (%)	77 (44.3)	49 (92.5)	28 (23.14)	0.001
Stage IV Disease	100 (57.5)	17 (32.1)	83 (68.6)	<0.001
Presence of Brain Metastasis	44 (25.3)	7 (13.2)	37 (30.6)	0.015
Presence of Bone Metastasis	73 (42)	9 (17)	64 (52.9)	<0.001
Absence of RT-Related Toxicity	135 (77.6)	42 (79.2)	93 (76.9)	0.728
Absence of Hematological Toxicity	143 (82.2)	45 (84.9)	98 (81)	0.535
Compliance with Radiotherapy	157 (90.2)	49 (92.5)	108 (89.3)	0.513
Absence of Hospitalization During RT	117 (67.2)	45 (84.9)	72 (59.5)	0.001
Systemic Therapy Administered	142 (81.6)	50 (94.3)	92 (76)	0.004
Surgical Treatment Performed	29 (16.7)	14 (26.4)	15 (12.4)	0.022
Purpose of Radiotherapy: Palliative	100 (57.5)	17 (32.1)	83 (68.6)	<0.001
Time to Post-RT Local Recurrence, days	129.5 (9–1323)	455 (37–1323)	97 (9–681)	<0.001
Time to Post-RT Distant Metastasis, days	146.5 (3–1323)	549 (25–1323)	100 (3–848)	<0.001
Number of RT Fractions	10 (1–35)	30 (1–33)	10 (1–35)	<0.001
Dose per RT fraction	300 (180–2100)	200 (180–2100)	300 (180–2100)	0.010
Total RT Dose, Gray	3000 (300–7000)	6000 (2000–6600)	3000 (300–7000)	<0.001
**Laboratory Parameters, median (min-max), mean ± standard deviation**				
Albumin, g/dL	39 (26–49)	42 (26–49)	38 (26–47)	<0.001
Creatinine, mg/dL	0.88 (0.37–6.42)	0.87 (0.6–1.4)	0.88 (0.37–6.42)	0.410
C-reactive protein, mg/L	26.37 (0.6–257.34)	7.53 (0.6–167)	30.16 (0.6–257.34)	0.010
Hemoglobin, g/dL	12.48 ± 1.9	12.9 ± 1.79	12.29 ± 1.92	0.052
White blood cell, 10^3^/µL	9.21 ± 2.9	9.23 ± 2.76	9.2 ± 2.96	0.95
Platelet, 10^3^/microL	282 (78–625)	281 (136–625)	283 (78–610)	0.775
Neutrophil count, 10^3^/µL	6 (0.86–14.39)	5.85 (1.32–12.55)	6.24 (0.86–14.39)	0.175
Lymphocyte, 10^3^/microL	1.74 (0.2–4.5)	2.21 (0.44–4.32)	1.52 (0.2–4.5)	<0.001
Monocyte count, 10^3^/microL	0.79 (0.03–1.87)	0.8 (0.31–1.49)	0.79 (0.03–1.87)	0.514
**Median follow-up time**				
Follow-up duration since initial diagnosis, months	12 (0–69)	25 (10–69)	7 (0–53)	<0.001
Follow-up duration since RT, months	8 (0–43)	22 (8–43)	4 (0–34)	<0.001

NSCLC, non-small cell lung cancer; ECOG: Eastern Cooperative Oncology Group, BMI: Body Mass Index.

**Table 2:** Comparison of Nutritional and Inflammatory Indices by Mortality status in the study population.

Parameters	Total (*n* = 174)	Survivors (*n* = 53)	Non-Survivors (*n* = 121)	*p*-Value
NLR	3.38 (0.42–44.05)	2.58 (0.57–20.24)	3.75 (0.42–44.05)	<0.001
PLR	159.74 (39.01–953.13)	120.85 (56.46–609.09)	168.03 (39.01–953.13)	0.002
MLR	0.44 (0.04–2.09)	0.37 (0.14–1.32)	0.48 (0.04–2.09)	0.011
ALI	282.37 (14.26–2835.07)	369.6 (36.84–1765.09)	233.16 (14.26–2835.07)	<0.001
SII	946.06 (92.06–10,922.81)	730.88 (143.33–5546.29)	1139.09 (92.06–10,922.81)	0.004
HALP	3.1 (0.29–13.73)	4.48 (0.58–9.85)	2.82 (0.29–13.73)	<0.001
CALLY	0.26 (0.01–13.65)	0.87 (0.02–13.65)	0.16 (0.01–4.44)	0.002
PNI	47.72 ± 7.69	51.84 ± 7.14	45.91 ± 7.24	<0.001
GNRI	102.93 ± 10.93	108.95 ± 10.77	100.29 ± 9.96	<0.001

**Table 3:** The Cox regression analyses evaluated the associations between immunonutritional indices and mortality in both univariable (crude) and adjusted models (separate regression models were designed for each index).

Parameters	For Mortality—Unadjusted HRs (CI)	*p*-Value	For Mortality—Adjusted HRs (CI) *	*p*-Value
NLR	1.055 (1.027–1.083)	<0.001	1.042 (1.010–1.074)	0.008
PLR	1.002 (1.001–1.004)	<0.001	1.002 (1.001–1.004)	<0.001
MLR	2.986 (1.767–5.045)	<0.001	3.027 (1.699–5.393)	<0.001
ALI	0.999 (0.998–1.000)	0.005	1.000 (0.999–1.000)	0.151
SII	1.021 (1.010–1.032)	<0.001	1.016 (1.003–1.029)	0.014
HALP	0.814 (0.737–0.899)	<0.001	0.844 (0.765–0.931)	<0.001
GNRI	0.952 (0.935–0.970)	<0.001	0.951 (0.928–0.974)	<0.001
PNI	0.931 (0.909–0.953)	<0.001	0.940 (0.917–0.964)	<0.001
CALLY	0.606 (0.414–0.887)	0.010	0.664 (0.465–0.949)	0.025

Abbreviations: NLR, neutrophil-to-lymphocyte ratio; PLR, platelet-to-lymphocyte ratio; MLR, monocyte-to-lymphocyte ratio; ALI, advanced lung cancer inflammation index; SII, systemic immune-inflammation index; SII was included in the Cox regression model by scaling per 100 units (SII/100), to improve the interpretability of its effect estimates; HALP = hemoglobin, albumin, lymphocyte, and platelet; CALLY, C-reactive protein–albumin–lymphocyte index; PNI, prognostic nutrition index; GNRI, geriatric nutrition risk index. * All indices were adjusted for age, body mass index (BMI), sex, type of lung cancer (SCLC and NSCLC), and presence of metastasis as covariates. The multicollinearity among these covariates was assessed using the variance inflation factor and tolerance values.

## Data Availability

Data available upon reasonable request from the corresponding author.

## Abbreviations

LC	lung cancer
RT	radiotherapy
NLR	neutrophil-to-lymphocyte ratio
PLR	platelet-to-lymphocyte ratio
MLR	monocyte-to-lymphocyte ratio
ALI	advanced lung cancer inflammation index
SII	systemic immune-inflammation index
HALP	hemoglobin–albumin–lymphocyte–platelet score
CALLY	C-reactive protein–albumin–lymphocyte index
PNI	prognostic nutritional index
GNRI	geriatric nutritional risk index
BMI	body mass index
OS	overall survival
ECOG	Eastern Cooperative Oncology Group
SCLC	small-cell lung cancer
SCC	squamous cell carcinoma
